# Tongue Shape Complexity in Children With and Without Speech Sound Disorders

**DOI:** 10.1044/2023_JSLHR-22-00472

**Published:** 2023-06-02

**Authors:** Marie Dokovova, Ellie Sugden, Gemma Cartney, Sonja Schaeffler, Joanne Cleland

**Affiliations:** a School of Psychological Sciences and Health, University of Strathclyde, Glasgow, United Kingdom; b School of Health Sciences, Western Sydney University, Richmond, New South Wales, Australia; c School of Health Sciences, Queen Margaret University, Edinburgh, United Kingdom

## Abstract

**Purpose:**

This study investigates the hypothesis that younger speakers and speakers with more severe speech sound disorders are more likely to use simpler (undifferentiated) tongue gestures due to difficulties with, or immaturity of, lingual motor control.

**Method:**

The hypothesis is tested using cross-sectional secondary data analysis of synchronous audio and high-speed ultrasound recordings from children with idiopathic speech sound disorders (*n* = 30, aged 5;0–12;11 [years;months]) and typically developing children (*n* = 29, aged 5;8–12;10), producing /a/, /t/, /ɹ/, /l/, /s/, and /ʃ/ in an intervocalic /aCa/ environment. Tongue shape complexity is measured using NINFL (Number of INFLections) and modified curvature index (MCI) from splines fitted to ultrasound images at the point of maximal lingual gesture. Age, perceived accuracy, and consonant are used as predictors.

**Results:**

The results suggest that as age increases, children with speech sound disorders have lower MCI compared to typically developing children. Increase in age also led to decrease of MCI for the typically developing group. In the group of children with speech sound disorders, perceptually incorrect /ɹ/ productions have lower MCI than correct productions, relative to /a/.

**Conclusions:**

There is some evidence of systematic tongue shape complexity differences between typically developing children and children with speech sound disorders when accounting for increase in age. Among children with speech sound disorders, increase in age and perceptually incorrect consonant realizations are associated with decreasing tongue shape complexity.

Most English-speaking children acquire all English consonants by the age of 7 years. At this stage, they should be also fully intelligible to unfamiliar listeners (Hustad et al., 2021; McLeod & Crowe, 2018). When a child's speech sound development diverges from the typically observed milestones and phonological processes, the term *speech sound disorder* is frequently used as a diagnostic umbrella term. While the prevalence of speech sound disorders among school-age children is about 3.6%–5% (Law et al., 2000; Shriberg et al., 1999; Wren et al., 2016), there is still no clear understanding of the causal link between the etiology of most speech sound disorders and their speech manifestation (Namasivayam et al., 2020). It has been argued that the traditional separation into phonetic and phonological speech sound disorders may not be an accurate reflection of the underlying speech sound production, as there might be a motor component in many of the speech sound difficulties that are perceived as complete sound deletions or substitutions (Cleland et al., 2017; Kabakoff et al., 2021; Namasivayam et al., 2020).

## Relationship Between Speech Sound Disorder Diagnosis and Tongue Shape Complexity

A motor component in speech sound disorders may affect “lingual differentiation,” the ability to move parts of the tongue independently (Gibbon, 1999). This ability has been shown to develop with children's maturation (Fletcher, 1989), and it underlies the accurate production of a number of speech sounds (Kabakoff et al., 2023). Hence, lingual differentiation during speech can be used as an index of motor maturation. Based on electropalatography and electromagnetic articulography research, Gibbon (1999) has estimated that 71% of children between 4 and 12 years of age with articulatory or phonological disorders have undifferentiated lingual gestures, compared to children with typical development. Undifferentiated lingual gestures occur when, instead of controlling the parts of the tongue independently, the tongue moves as one body, potentially coupled with the jaw. This likely reflects a motor immaturity and can lead to perceived homophony in the child's sound inventory. Gibbon's (1999) estimates are illustrative of more severe or persistent cases of speech sound disorder, as they are based on a sample of 17 children referred for electropalatography treatment for persistent speech sound difficulties.

Detecting undifferentiated lingual gestures requires instrumental articulatory techniques. In Gibbon's (1999) electropalatography figures, the undifferentiated lingual gesture is clearly visible as a substantial amount of tongue–palate contact during coronal and dorsal articulations. Undifferentiated lingual gestures can also be detected using ultrasound tongue imaging. Cleland et al. (2017) and Cleland and Scobbie (2021) describe undifferentiated tongue shapes in children with persistent velar fronting using ultrasound tongue imaging. However, in both studies, the undifferentiated lingual gestures were hard to quantify with existing measures of tongue shape. A recent comparison of ultrasound tongue imaging quantitative measures has identified two measures that reliably capture small differences in tongue shape complexity in the speech of adults and typically developing children (Kabakoff et al., 2023). One of the measures is NINFL (Number of INFLections of the tongue; Preston et al., 2019). It is derived from a tracing of the tongue contour in a midsagittal view and sums the number of convex and concave curves that exceed a specific threshold. Another measure, the modified curvature index (MCI), is calculated by integrating the unsigned curvature across points along the tongue contour and can also effectively capture consonant-related tongue shape complexity differences in children's speech (Dawson et al., 2016; Kabakoff et al., 2023).

If a child's tongue shape complexity is lower than that of their peers, this might signal potential difficulties or immaturity with speech motor control or presence of undifferentiated lingual gestures. Therefore, the NINFL and MCI measures of tongue shape complexity have the potential to identify the relative contribution of motor program deficits in speech sound disorders and contribute to the intervention decision-making process (Kabakoff et al., 2021). However, considerations such as speaker age, consonant complexity, and diagnosis need to be taken into account when using these relatively recent measures. Previous research has provided preliminary evidence regarding these factors.

## Relationship Between Perceptual Accuracy and Tongue Shape Complexity

Kabakoff et al. (2021) use NINFL and MCI to compare lingual differentiation in younger children (ages 4–6 years) with and without speech sound disorder across a range of consonants. They report that typically developing children have higher NINFL for /ɹ/ than children with speech sound disorder. This finding is similar to the NINFL investigation of Preston et al. (2019) and the qualitative study of Klein et al. (2013). In addition, in the work of Kabakoff et al. (2021), higher perceptual ratings of accuracy for /ɹ/ were also associated with higher NINFL across typically developing children and children with speech sound disorder. There was no effect of perceptual accuracy or diagnosis on the complexity of other sounds such as /k/, /l/, /j/, and /w/. As in the work of Preston et al. (2019), NINFL was found to be more sensitive to perceptual accuracy, diagnosis, and age differences than MCI. However, recent quantitative studies suggest that MCI measurements are better aligned with theoretical predictions than NINFL in typically developing younger children (Kabakoff et al., 2023) and older children with speech sound disorder (Kabakoff et al., 2022). Of these two studies, Kabakoff et al. (2022) investigated the relationship between the complexity of /ɹ/ and accuracy, although accuracy was acoustically measured as the distance between F2 and F3, not perceptually. They found that higher tongue shape complexity was linked to lower accuracy before treatment and with higher accuracy after treatment. The unexpected result before treatment was accounted for as a potential maladaptive outcome from the children's previous treatments.

Other research also shows that lower lingual complexity is not always linked with low accuracy. Gibbon (1999) discusses reports of children whose alveolar consonant targets were variably transcribed as correct, or as exhibiting backing to what was perceived as a velar or palatal place of articulation. Electropalatography investigations revealed that these three perceptual variants involved the same undifferentiated lingual gesture. The perceptual difference was caused by the part of the tongue that was released last from the palate.

The evidence suggests that the presence of speech sound disorder and decreased perceptual accuracy may sometimes be associated with lower tongue shape complexity, across a variety of measures. However, the majority of relevant ultrasound data are based on the American English /ɹ/ (Kabakoff et al., 2021, 2022; Klein et al., 2013; Preston et al., 2019). While /ɹ/ is a common target for speech and language therapy intervention in North America (Hitchcock et al., 2015), it is not usually targeted in the United Kingdom, where a large variety of rhotic articulations are considered part of the dialectal diversity (King & Ferragne, 2020). For example, in Scottish English, /ɹ/ in non coda positions is most commonly realized as an approximant (tip-up, bunched, and retroflex), which are also found in U.S. varieties of English, with occasional tap realizations medially or in trochaic positions (Lawson, 2018, 2019; Scobbie et al., 2006). Trill realizations are rare and associated with older speakers (Lawson, 2018; Scobbie et al., 2006). According to Lawson et al.'s (2019) ultrasound study, differences between Scottish English and North American realizations of /ɹ/ can be found in the timings but not in the fundamental pattern of the gestures.

By also including non rhotic consonants, this article aims to increase the clinical relevance of tongue shape complexity research to speech and language therapist practitioners beyond the United States. This study addresses the question of whether speech sound disorder diagnosis and perceptual accuracy on tongue shape complexity can be observed in a larger sample of speakers compared to previous research.

## Expected Tongue Shape Complexity Across Consonants

Articulatory imaging has been used to observe lingual differentiation, that is, the degree to which different parts of the tongue can move semi-independently from the rest (e.g., the tongue blade, the midsagittal portion of the tongue, and the lateral edges of the tongue; Farnetani & Recasens, 2010; Nguyen et al., 1996; Stone, 1991). Relative independence can be achieved because muscles insert in different parts of the tongue, resulting in different local contractions. For example, the genioglossus muscle inserts medially, while the hyoglossus and styloglossus insert laterally (Stone, 1991). As a result, the laterally inserting muscles can contract the lateral sides of the tongue and create convex and concave shapes in a cross-sectional view of the tongue, in addition to contributing to its raising and lowering, while the medially inserting genioglossus can create a midsagittal groove of the tongue (Stone, 1991). This possibility for differentiation allows the tongue to produce a number of shapes for the articulation of speech sounds.

Kabakoff et al. (2023) review five different hierarchies of consonant complexity, based on the lingual differentiation required, the observed error patterns, and the age of acquisition of the consonants (Crowe & McLeod, 2020; Dawson et al., 2016; Kent, 1992; Shriberg, 1993; Studdert-Kennedy & Goldstein, 2003). Despite some differences between the hierarchies, tongue shapes for voiceless stops and glides are consistently reported as less complex than fricatives and liquids. We could expect, accordingly, that motor program deficits or immaturity, related to speech sound disorders or age, are more likely to manifest as speech targets requiring more complex tongue shapes such as /ɹ/, /l/, /s/, or /ʃ/ compared to /t/.

When measured at the point of maximum contact,^1^ there are differences in how much lingual differentiation is needed across consonants. For instance, alveolar and velar stops differ in their primary place of articulation, but instrumental data show that their respective tongue shapes involve additional differentiation beyond what is suggested by their phonetic label. The alveolar stops /t/ and /d/ are usually produced with a constriction in the alveolar region of the palate and simultaneous lateral contact (Gibbon, 1999). The postalveolar and alveolar fricatives /ʃ/ and /s/ are produced with elevation of the tongue blade toward the postalveolar and alveolar part of the palate, respectively, based on ultrasound tongue imaging (Zharkova, 2013), with additional bracing of the lateral edges of the tongue, based on electropalatography imaging (Hardcastle et al., 1987). Zharkova (2013) illustrates typical adult and child productions of /ʃ/ and /s/ using examples from the database by Zharkova (2009). Midsagittal ultrasound images of /ʃ/ and /s/ suggest qualitatively that adult productions do not require additional differentiation, but the children's realizations had more inflections: in the back portion of the tongue for /s/ and with a groove in the midsection of the visible tongue contour for /ʃ/. According to Gibbon's (1999) review, these sibilants may be associated with backing and/or stopping errors in children with speech sound disorders, which would have impact on their midsagittal realizations. It should also be noted that there is a similarity in the realizations of /t/, /d/, and /s/. They all have lateral bracing and tongue blade elevation toward the alveolar region. Together, they would be expected to have simpler midsagittal complexity compared to /ʃ/ where the more posterior palatal or palato-alveolar stricture might lead to an inflection.

The consonants /l/ and /ɹ/ are considered among the most complex sounds in English, requiring more tongue inflections than other consonants (Studdert-Kennedy & Goldstein, 2003). /ɹ/ requires some movement of the lips, tongue tip, body, and dorsum, depending on the speaker's sociolinguistic variety of English (Adler-Bock et al., 2007; Lawson et al., 2013, 2018), while /l/ involves body elevation, tongue tip contact with the alveolar ridge, the teeth, or the palate (Lawson et al., 2018). Common errors for these consonants involve gliding, using /w/ or /j/, which require simpler articulatory gestures (Inkelas & Rose, 2008; Studdert-Kennedy & Goldstein, 2003). /w/ requires lip rounding and tongue dorsum elevation toward the velum (without the additional tongue tip approximations required by /ɹ/), and /j/ involves tongue blade or body approximation toward the palate (without the additional tongue tip contact with the alveolar ridge or teeth needed for /l/; Lawson et al., 2018). Hence, it is important to take the potential effect of consonant into consideration when studying the effects of age, speech sound disorder diagnosis, and severity on tongue shape complexity in speech.

## Expected Relationship Between Age and Tongue Shape Complexity

Speech motor control and the ability to coordinate movements are susceptible to change over the life span (Singh & Singh, 2008). One of the anatomical changes that occurs throughout childhood is that a person's relative tongue size reduces in proportion to the size of their vocal tract (Bosma, 1963; cited in Kabakoff et al., 2021). This allows space for finer lingual differentiation as a child grows: Electropalatographic recordings show that the number of palate sensors activated decreases with age, implying less tongue–palate contact (Fletcher, 1989). The laryngeal and hyoid descent, which progresses most rapidly during the first years of life and then again in puberty, also contributes to the gradual expansion of the oral cavity (Lieberman et al., 2001; Vorperian et al., 2009). Changes in dentition in school-age children are also linked to some differences in speech production (Pahkala et al., 1991). Unfortunately, a limitation of the ultrasound technique is that it does not provide direct information about the shape of the oral cavity and dentition, which can affect the resting tongue posture and the range of movement of the tongue (Brunner et al., 2009; Kotsiomiti & Kapari, 2000).

Namasivayam et al. (2020) review evidence that between-articulators coordination completes its development before within-articulator coordination, specifically the coordination within the tongue, which has multiple points of inflection. As discussed in the previous section, within-articulator coordination is particularly relevant for consonants that require higher level of lingual differentiation (like liquids), compared to consonants that require between articulator coordination (like alveolar stops). The different requirements for lingual differentiation explain some of the differences in consonant age of acquisition.

Children's typical error patterns also illustrate the gradual refinement of articulation skills. According to McLeod and Crowe (2018), liquids, affricates, sibilants, and interdental fricatives are among the later-acquired sounds (approximately between 3;6 [years;months] and 6 years of age). A typical phonological error pattern observed in children still acquiring these consonants is gliding of liquids (e.g., producing [ɫ] and [ɹ] as [w], and [l] as [j]; Bowen, 2007), which should involve a lower level of tongue shape complexity. For example, Namasivayam et al. (2020) explain stopping of fricatives as target overshoot and word-final devoicing and cluster simplification as simplification of gestural scores. These typical developmental errors decrease gradually, as children grow, indirectly suggesting that children are beginning to master the lingual gestures and differentiation required for correct consonant production (Bowen, 2007).

Based on the evidence of tongue size relative to the vocal tract and the gradual elimination of typical error patterns observed in younger speakers, it may be concluded that younger age would be associated with lower levels of tongue shape complexity. However, these predictions have not been directly confirmed using articulatory measurements. The qualitative observations of Kabakoff et al. (2023) reveal no differences in NINFL and MCI between adults and children for earlier-acquired sounds. However, they observed that adults have higher NINFL and MCI than children for later-acquired sounds, such as /l/ and /ɹ/, suggesting some evidence for tongue shape complexity maturation.

The only study that has quantified the effect of age in relation to NINFL and MCI, to our knowledge, is that of Kabakoff et al. (2021). They observed that younger children exhibit higher levels of tongue shape complexity for /t/ than older children, contrary to theoretical predictions. However, this study is better equipped to analyze the relationship between age and tongue shape complexity, as the sample includes a wider age range (5–12 years) than the study of Kabakoff et al. (2021; 4–6 years).

## Research Questions

This study investigates the relationship between tongue shape complexity for different consonants and the predictors: speaker age, the presence or absence of a speech sound disorder diagnosis, and its severity. The first research question aims to replicate the finding of less tongue shape complexity (NINFL or MCI) in children with speech sound disorders compared to typically developing children for consonants /ɹ/, /l/, /ʃ/, and /s/ but not for /t/. This was previously reported in the works of Klein et al. (2013), Preston et al. (2019), and Kabakoff et al. (2021). Of these studies, that of Kabakoff et al. (2021) is the only study that investigated additional consonants to /ɹ/. They observed an effect of diagnosis only for /ɹ/ but not for the other consonants. This study expands on previous research by focusing on Scottish English and by analyzing additional consonants to those investigated in the other child-data studies (Kabakoff et al., 2021; Klein et al., 2013; Preston et al., 2019).

The second research question focuses on whether older children produce more lingually complex sounds than younger children, especially for consonants /ɹ/, /l/, /ʃ/, and /s/ but not as much for /t/. Evidence for the effect of age on tongue shape complexity in children is limited to the findings of Kabakoff et al. (2021), which contradicts the theoretical expectations that an increase in age would be associated with increased tongue shape complexity. This study includes a wider age range of speakers than the study of Kabakoff et al. (2021) and is therefore more equipped to detect the effect of age on tongue shape complexity.

The final research question investigates whether incorrect productions of consonants /ɹ/, /l/, /ʃ/, /s/, and /t/ have a decrease in NINFL and MCI. Kabakoff et al. (2021) and the post-therapy results from Kabakoff et al. (2022) report increased NINFL and MCI, respectively, for more accurate /ɹ/.

## Method

### Participants

Two groups of children participated in this study: those with diagnosed speech sound disorders and those without. Data from both groups were sourced from the UltraSuite corpus (Eshky et al., 2018): Ultrax Typically Developing and ULTRAX2020.

Thirty children with typical development consented to participate. All participants had Scottish accents. One participant had a 91 percentage consonants correct score on the Diagnostic Evaluation of Articulation and Phonology (DEAP) diagnostic screen (Dodd et al., 2006) because of several labialized productions for /ɹ/ and /ʃ/ fronting. The participant's data were excluded from the analysis, leaving a total of 29 typically developing participants (15 girls, 14 boys), aged between 5;8 and 12;10 (*M* = 9;8, *SD* = 2). The other participants all scored within normal limits on the DEAP diagnostic screen (Dodd et al., 2006). With two exceptions (with standard scores of 72 and 76), all participants had British Picture Vocabulary Scale (Dunn, 2009) standard scores within normal limits (overall mean = 103, *SD* = 14). The speech of all typically developing children whose data were kept for analyses was within norm for their age. The demographic information of each typically developing participant is presented in Appendix A.

The participants with speech sound disorders are described in a previous publication (Sugden & Cleland, 2022). The clinical data were collected as part of the ULTRAX2020 project, which aimed to obtain and analyze ultrasound data from children with different speech sound disorder subtypes, presenting in community clinics in Scotland (Cleland et al., 2018). As a result, the complete diagnostic information for the participants was available to the community clinicians but not to the researchers.

The participants attended speech and language therapy services in the east of Scotland. The inclusion criteria were as follows: aged between 5 and 16 years with a diagnosis of any speech sound disorder of unknown origin, given by their speech and language therapist. The exclusion criteria were as follows: current or repaired cleft lip and/or palate, either syndromic or not; no spoken English; evidence of severe or profound hearing loss; and evidence of severe or profound learning disability. Other concomitant communication disorders were not excluded. Eligibility was monitored by their speech and language therapist. Six additional participants that were not reported in the work of Sugden and Cleland (2022) are included in this study because their data were collected later. The same data collection protocol was followed.

There were 32 children with speech sound disorders who initially consented to participate in the project and whose parents also gave consent. The data sets of two children were not included for analysis because they had withdrawn consent during the recording, leaving a total of 30 children (22 boys and eights girls) for analysis. They were aged 5;0–12;11 (*M* = 8;2, *SD* = 2). Of those children, 16 were reported to have articulation disorder, three have motor speech disorder, and three have phonological disorder. Of the remaining eight children, one had residual speech sound errors, one had inconsistent phonological disorder, one was still undergoing a diagnostic process, for one there was no report, and the remaining children had inconclusive diagnoses (e.g., query childhood apraxia of speech). The child with residual speech sound errors, aged 8;11, had been previously treated for anterior ankyloglossia, but there is no other information suggesting potential anatomical differences in this group of children with speech sound disorders of unknown origin. The demographic information of each participant with speech sound disorders is presented in Appendix B.

### Speech Recording Materials

The speech materials are a subset of those described in the work of Cleland et al. (2018). This study focuses on the English sonorants /l/ and /ɹ/ and voiceless obstruents /ʃ/, /s/, and /t/ in an /aCa/ context. The first vowel /a/ from /apa/ syllables was chosen as a reference against which the other consonants' tongue shape complexity was compared, in line with Kabakoff et al. (2021), who used /æ/. The /a/ phoneme best represents the Scottish accent low front vowel.

The remaining consonants (apart from /t/) were chosen because, as discussed in an earlier section, they are consistently categorized as requiring complex tongue shapes across different hierarchies (Kabakoff et al., 2023). As a result, they are suitable candidates to investigate the presence of lingual differentiation immaturity. The consonant /t/ was chosen, first, to investigate if the age-related decline in complexity reported in the work of Kabakoff et al. (2021) can be replicated. Second, unlike the rest of the consonants, /t/ is usually classified as an early-acquired sound, requiring a less differentiated gesture. It is also produced with a tongue tip stricture in the alveolar area, making it comparable to the other consonants under investigation (alveolar and postalveolar).

The children with speech sound disorders repeated each consonant in an /aCa/ environment 10 times, and the typically developing children produced it only once. All children heard the same prerecorded model production of the target syllable before producing it. The model productions were by the fifth author who is a native and current speaker of Scottish English.

### Data Collection and Ethical Considerations

The typically developing data collection project received ethical approval from the National Health Service Ethics Research Committee (Eshky et al., 2018), and the speech sound disorder data collection project received ethical approval from the South East Scotland Research Ethics Committee 1 (Sugden & Cleland, 2022). An ethical approval for the secondary data analysis was provided from [redacted 1] Prof. James M. Scobbie, the ethics representative of the [redacted 1] Prof. James M. Scobbie (reference 2022.01.31.MS.SS).

### Ultrasound Recordings

The details of the ultrasound recording setup have also been reported in the work of Eshky et al. (2018) for the typically developing group and in the work of Sugden and Cleland (2022) for the speech sound disorder group. Typically developing children were recorded in an ultrasound research laboratory on the campus of Queen Margaret University using a Sonix RP machine as part of the UltraSuite corpus by speech and language therapists (Eshky et al., 2018). Children with speech sound disorders were recorded in quiet rooms in the community clinics, facilitated by the local speech and language therapists who were trained in the procedures. The speech sound disorder recordings were carried out using an ultrasound Micro machine, operated on a laptop via SonoSpeech (Version 2.17.10, Version 2.18.01, Version 2.18.02, or Version 2.18.04; Articulate Instruments Ltd., 2019). This specialized software was designed to collect synchronized ultrasound and audio recordings from speech and language therapy sessions.^2^

Recordings with typically developing children were obtained at approximately 120 fps with a 134° field of view. Speech sound disorder recordings were obtained at approximately 100 fps, with a 119°–162° field of view. The microconvex ultrasound probe (5–8 HHz, 10‐mm radius) was stabilized under the speaker's chin using an UltraFit lightweight plastic headset for the children with speech sound disorders, keeping the same angle of the probe relative to the speaker's mandible throughout the recording. For the typically developing children, the probe was stabilized using a metal headset. The two headsets have been shown to be similar in systematic comparisons (Pucher et al., 2020). The headset and probe were fitted by experienced speech and language therapist clinicians or researchers who carried out the recordings, and they qualitatively examined the fit to ensure that alignment was achieved. However, a limitation of this method is that there are no objective ways to verify their judgment and to ensure that the probe alignment remained constant throughout the recording.

### Analyses

#### Spline Fitting

Splines were fitted at the point of maximal lingual gesture, using Articulate Assistant Advanced software (Articulate Instruments Ltd., 2012). The frame with the maximal lingual gesture was identified manually by the third author, a qualified speech and language therapist. First, using the audio, waveform, and spectrogram, a range of ultrasound frames of interest were identified. The third author also listened to each sound and labeled it as correct or incorrect based on auditory judgment. A reliability check was performed on the accuracy results of six of the participants with speech sound disorders (20% of the participants with speech sound disorders, or 250 consonant tokens) by using the auditory judgments of another qualified speech and language therapist who provided transcriptions for Sugden and Cleland (2022). Cohen's κ was 0.79, indicating substantial agreement.

For stops, the frame of the maximal lingual gesture consisted of the consonant closure phase, and for continuants, the whole duration of the consonant was annotated. The tongue movements in these regions of interest were observed frame by frame. When the frame with maximal lingual gesture was identified, an annotation was made to save the time point. For /a/, a frame was extracted from the vowel midpoint. The beginning and end of the vowel were identified using the beginning and end of the periodic waveform, as primary indicators, and the beginning and end or shift in spectrogram formants as a secondary feature. The midpoint was identified automatically by the Articulate Assistant Advanced software. A spline was fitted to indicate the surface of the tongue using semiautomatic edge detection from the Articulate Assistant Advanced software, which has recently been validated as a reliable method for tracking ultrasound tongue images (Roon et al., 2022). Each of the splines in all the data sets was inspected qualitatively, and if small corrections were required, they were retraced manually; the manual tracing was additionally corrected using the Articulate Assistant Advanced “snap-to-fit” function. A spline was fitted for each of the repetitions of each consonant. Although the children were instructed to produce 10 repetitions, some children occasionally produced fewer repetitions, resulting in 1,694 tokens to which splines were fitted. In addition to that, some data had to be excluded (see following section). The statistical analysis, described in a following section, is not impeded by unequal numbers of tokens per consonant.

#### Extraction of NINFL and MCI

Once the tongue splines were fitted, NINFL measures were automatically extracted for each of the consonants from every speaker and exported to a .csv file. The software Articulate Assistant Advanced (Articulate Instruments Ltd., 2012) uses Preston et al.'s (2019) procedure for calculating NINFL, transferred from MATLAB to Delphi (personal correspondence with Wrench). The original MATLAB procedure is available at https://osf.io/xzdb7/.

NINFL is calculated with reference to the curvature of a tongue spline. For every point along the tongue spline, the curvature at that point is the reciprocal of the radius of a circle that best conforms to the tongue curve at that given point (Britannica, 2013). NINFL is the count sum of all changes of the sign of the curvature (e.g., from positive to negative) when the curvature is different from zero. The curvature is trimmed, so that small values are not included. Small values are those where the associated radius is smaller than 0.3 multiplied by the distance along the curve from the first to the last point. The trim threshold value of 0.3 was heuristically chosen (Preston et al., 2019).^3^ Retroflex points are also trimmed. When the procedure was implemented in Articulate Assistant Advanced, the splines were smoothed using a 6-point average smoothing function. In addition, all inflections occurring in the first and last 5% of the contour were disregarded due to high number of false detections.

In summary, a change from a concave to a convex tongue shape is considered an inflection and the sum of these inflections is captured by NINFL (Kabakoff et al., 2021). When the NINFL formula was adapted to Articulate Assistant Advanced, all output assumes the presence of one curve along the tongue spline, so an output of 0 is equivalent to 1 in the original formula. Hence, before the data were analyzed, 1 was added to all NINFL measurements. Following the approach used in the works of Preston et al. (2019) and Kabakoff et al. (2021), all NINFL values over 5 were filtered out (*n* = 74). These filtered-out tokens were also not included in the MCI analysis.

The MCI for each consonant token was calculated using the procedure and python script provided in the work of Dawson et al. (2016). The procedure requires input of the *x* and *y* Cartesian coordinates of the tongue spline, which are used to calculate the absolute curvature at each equidistant point along the spline, and those values are then integrated (Kabakoff et al., 2023). Following Dawson et al. (2016), all resulting values over 6 were excluded from all analyses (*n* = 39).

#### Statistical Analyses

Two statistical models were run to answer the first and second research questions about the difference in tongue shape complexity between children with and without speech sound disorders and across ages. Tongue shape complexity is operationalized as NINFL and MCI to compare the sensitivity of the complexity measures to these predictors. The data were wrangled and visualized using the “tidyverse” package (Wickham et al., 2019).

To analyze NINFL, ordinal mixed-effects regressions were used, following the approach described in the work of Kabakoff et al. (2021), using the “clmm” package (Christensen, 2015) in R Studio (RStudio Team, 2021). The outcome variable NINFL was coded as an ordinal variable with values ranging between 1 and 5. An ordinal mixed model was run with a categorical predictor diagnosis (treatment coded with reference typically developing vs. speech sound disorders) and phoneme (deviation coded, /a/ as reference, compared to /l/, /ɹ/, /ʃ/, /s/, and /t/) and continuous predictor age (months, centered). Deviation coding allows the effects of the other variables to be considered across phonemes, as opposed to with reference to only one phoneme. The interaction between age and diagnosis was included to investigate whether the increase of age was associated with increased tongue shape complexity across consonants, differentially for each group. The interaction between age and each of the phoneme contrasts was included to investigate if typically developing speakers' tongue shape complexity changes for each of the consonants with the increase of age. The interaction between diagnosis and each of the phoneme contrasts was included to check if, keeping age stable, there is a difference in the tongue shape complexity of children with and without speech sound disorders for each of the consonants, replicating results from the work of Kabakoff et al. (2021). By-participant random intercepts were included to account for speaker-specific variability in complexity, and by-participant random slopes for phoneme were included to account for variability in how each speaker produces each phoneme. A linear mixed model was run with the same structure as the model above but with MCI as a categorical outcome variable (Bates et al., 2015). A three-way interaction was not included.

The third research question focuses on the relationship between perceptual accuracy and tongue shape complexity for children with speech sound disorders. To address this question, two statistical models were fitted to the subset of data from children with speech sound disorders only. An ordinal mixed-effects model was used with NINFL as an outcome variable and the following predictors: error (treatment coded, correct as reference vs. error), phoneme (deviation coded to compare /a/ to each of the consonants), and their interaction. Age (in months, centered) and the interaction between age and error, and age and phoneme were included to control for the potential effect of increased tongue shape complexity with age. By-participant random intercepts were included to account for speaker-specific variability in complexity, and by-participant random slopes for phoneme and error were included to account for variability in how each speaker produces each phoneme and the difference between erroneous and correct productions. A linear mixed model was run with the same structure as the model above but with MCI as a continuous outcome variable. A three-way interaction was not included.

## Results

### Relationship Between a Speech Sound Disorder Diagnosis and Tongue Shape Complexity

The first research question focuses on whether a speech sound disorder diagnosis is associated with lower tongue shape complexity compared to no diagnosis. The results of the ordinal mixed model with NINFL and the linear mixed model with MCI are summarized in Table 1.

**Table 1.** Results of the model comparing NINFL (top) and MCI (bottom) between TD children and children with SSDs across ages and consonants.

NINFL TD vs. SSDs					
Predictor	Estimate		*SE*	*z* value	*p* value
Age	< 0.01		0.01	0.18	.855
Diagnosis (TD vs. SSDs)	−0.44		0.27	−1.63	.104
Phoneme /a/ vs. /l/	0.78		0.57	1.35	.176
Phoneme /a/ vs. /ɹ/	2.36		0.50	4.69	< .001*
Phoneme /a/ vs. /ʃ/	0.04		0.50	0.08	.941
Phoneme /a/ vs. /s/	−1.53		0.51	−2.99	.003*
Phoneme /a/ vs. /t/	−1.60		0.49	−3.25	.001*
Age: diagnosis (TD vs. SSD)	0.01		0.01	0.87	.384
Diagnosis (TD vs. SSD): phoneme /a/ vs. /l/	−0.94		0.83	−1.14	.253
Diagnosis (TD vs. SSD): phoneme /a/ vs. /ɹ/	−0.89		0.72	−1.24	.214
Diagnosis (TD vs. SSD): phoneme /a/ vs. /ʃ/	0.29		0.72	0.40	.690
Diagnosis (TD vs. SSD): phoneme /a/ vs. /s/	0.83		0.72	1.14	.253
Diagnosis (TD vs. SSD): phoneme /a/ vs. /t/	0.58		0.70	0.83	.405
Age: phoneme /a/ vs. /l/	−0.02		0.02	−0.95	.343
Age: phoneme /a/ vs. /ɹ/	< 0.01		0.02	0.23	.816
Age: phoneme /a/ vs. /ʃ/	< 0.01		0.02	0.01	.991
Age: phoneme /a/ vs. /s/	< 0.01		0.02	0.16	.875
Age: phoneme /a/ vs. /t/	< 0.01		0.02	0.43	.667
**MCI TD vs. SSDs**					
**Predictor**	**Estimate**	***SE***	***df***	***t* value**	***p* value**
(Intercept)	3.52	0.07	65.13	51.16	< .001*
Age	−0.01	0.00	59.87	−2.33	.023*
Diagnosis (TD vs. SSD)	−0.01	0.10	64.67	−0.14	.890
Phoneme /a/ vs. /l/	0.11	0.18	78.45	0.60	.552
Phoneme /a/ vs. /ɹ/	0.54	0.18	90.48	3.03	.003*
Phoneme /a/ vs. /ʃ/	0.52	0.17	73.04	3.05	.003*
Phoneme /a/ vs. /s/	−0.42	0.15	109.29	−2.74	.007*
Phoneme /a/ vs. /t/	−0.62	0.17	77.10	−3.67	< .001*
Age: diagnosis (TD vs. SSD)	−0.01	0.00	65.58	−2.14	.036*
Diagnosis (TD vs. SSD): phoneme /a/ vs. /l/	−0.32	0.26	77.92	−1.23	.224
Diagnosis (TD vs. SSD): phoneme /a/ vs. /ɹ/	−0.08	0.26	88.72	−0.32	.749
Diagnosis (TD vs. SSD): phoneme /a/ vs. /ʃ/	−0.26	0.25	72.29	−1.05	.296
Diagnosis (TD vs. SSD): phoneme /a/ vs. /s/	−0.02	0.22	107.28	−0.08	.936
Diagnosis (TD vs. SSD): phoneme /a/ vs. /t/	0.26	0.24	76.88	1.08	.282
Age: phoneme /a/ vs. /l/	0.01	0.01	45.11	0.91	.369
Age: phoneme /a/ vs. /ɹ/	0.01	0.01	58.27	0.80	.429
Age: phoneme /a/ vs. /ʃ/	−0.01	0.01	49.19	−1.44	.156
Age: phoneme /a/ vs. /s/	< 0.01	0.01	48.11	−0.56	.581
Age: phoneme /a/ vs. /t/	< 0.01	0.01	44.93	0.43	.667

*Note.* Results for predictors that are significant at the *p* < .05 level are bolded and marked with an asterisk. NINFL = Number of INFLections; MCI = modified curvature index; TD = typically developing; SSDs = speech sound disorders; *SE* = standard error; *df* = degrees of freedom.

**Figure 1.**
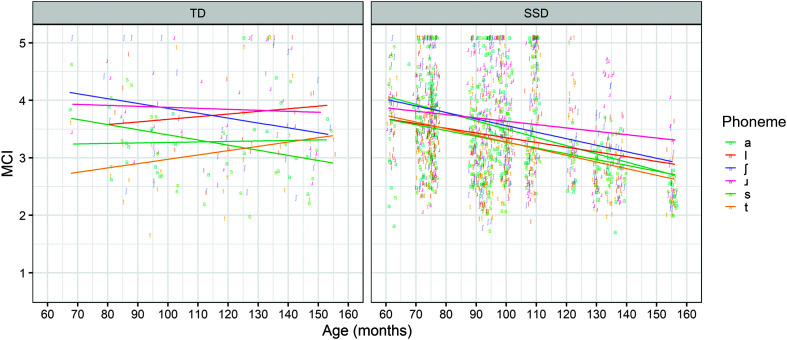
Boxplot showing modified curvature index (MCI) per consonant for typically developing (TD) children and children with speech sound disorders (SSDs), according to age (in months).

There were no significant effects of diagnosis in the NINFL model. In the MCI model, there was a significant interaction between age and diagnosis, such that children with speech sound disorders had an additionally lower MCI than typically developing children as age increased. This is illustrated in Figure 1. The prediction that children with speech sound disorders have lower tongue shape complexity than typically developing children was supported but only as age was increasing and only using MCI as a measure for complexity.

### Relationship Between Age and Tongue Shape Complexity

The second research question focused on whether there was a change in tongue shape complexity across consonants as age increased. According to the NINFL model results in Table 1, there were no significant effects of age. According to the MCI model results in Table 1, there was a significant effect of age, such that an increase in age for typically developing speakers across consonants was associated with a decline in MCI. In addition, as mentioned in response to the first research question, there was a significant interaction between age and diagnosis across consonants. The prediction that higher age is associated with higher level of tongue shape complexity for typically developing children was not supported, and instead, a negative relationship was observed.

**Figure 2.**
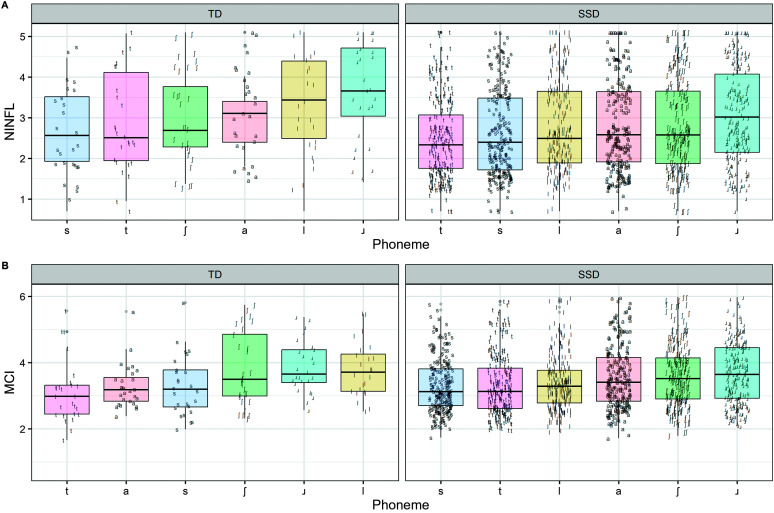
Plot showing the Number of INFLections (NINFL) and modified curvature index (MCI; *y*-axis) per consonant per group, typically developing (TD) and with speech sound disorders (SSDs), plotted according to increasing median value (*x*-axis).

In addition to addressing these research questions, the models provide information on differences in tongue shape complexity between consonants for typically developing children. Focusing on NINFL first, the results suggest that when keeping age constant for typically developing participants, /ɹ/ had significantly higher NINFL than /a/. Also, /s/ and /t/ had significantly lower NINFL than /a/. There were no other statistically significant effects. Focusing on MCI, /ɹ/ and /ʃ/ were significantly more complex than /a/ for typically developing speakers, keeping age constant, while /s/ and /t/ were significantly less complex than /a/. A depiction of the relative complexity of the phonemes is shown in Figure 2. These results are in line with some predictions that /ɹ/ and /ʃ/ require a relatively high level of tongue shape complexity, while /t/ does not. In addition, it was expected that the articulatory similarity /s/ and /t/ (with lateral bracing and alveolar stricture) might be associated with similarly lower tongue shape complexity than the more posterior /ʃ/, which is supported in Figure 2.

### Relationship Between Accuracy and Tongue Shape Complexity

**Figure 3.**
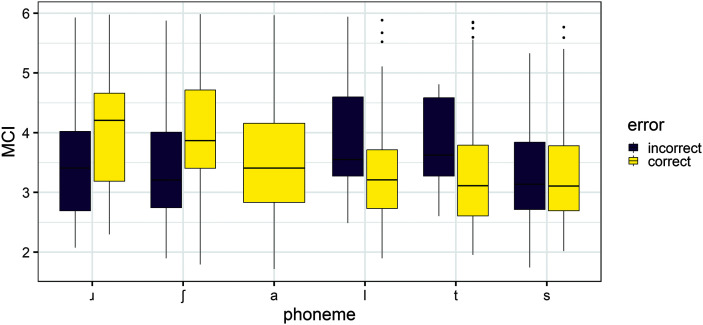
Boxplot showing the modified curvature index (MCI; *y*-axis) per consonant, according to accuracy for the group with speech sound disorders (SSDs).

The following results about the relationship between accuracy and tongue shape complexity are based only on the subset of children with speech sound disorders. The results of the NINFL model (see Table 2) suggest that there was no significant effect of error and no significant interactions between error and phoneme or error and age. The results of the MCI model (see Table 2) suggest that there was a significant interaction between error and the phoneme contrast between /a/ and /ɹ/. When perceptually incorrect, /ɹ/ had simpler lingual gesture than when produced correctly, relative to /a/. This supports the hypothesis that erroneous productions have less differentiated lingual gestures but was only found for one consonant and one complexity measure (MCI). This is illustrated in Figure 3.

**Table 2.** Results of model comparing NINFL (top) and MCI (bottom) across different consonants for children with SSDs with different PCC and different ages.

NINFL TD vs. SSDs					
Predictor	Estimate		*SE*	*z* value	*p* value
Age	0.01		0.01	1.12	.263
Error (correct vs. error)	−1.05		1.70	−0.62	.538
Phoneme /a/ vs. /l/	−0.45		0.59	−0.76	.446
Phoneme /a/ vs. /ɹ/	2.81		0.81	3.47	.001*
Phoneme /a/ vs. /ʃ/	0.90		0.79	1.14	.255
Phoneme /a/ vs. /s/	−1.29		0.76	−1.70	.088
Phoneme /a/ vs. /t/	−1.54		0.53	−2.88	.004*
Age: error (correct vs. error)	−0.02		0.02	−0.95	.344
Error (correct vs. error): phoneme /a/ vs. /l/	1.03		3.41	0.30	.762
Error (correct vs. error): phoneme /a/ vs. /ɹ/	−0.70		3.59	−0.20	.845
Error (correct vs. error): phoneme /a/ vs. /ʃ/	0.58		3.40	0.17	.864
Error (correct vs. error): phoneme /a/ vs. /s/	1.19		3.40	0.35	.727
Error (correct vs. error): phoneme /a/ vs. /t/	−0.03		0.03	−1.27	.203
Age: phoneme /a/ vs. /l/	0.00		0.03	−0.13	.899
Age: phoneme /a/ vs. /ɹ/	0.01		0.03	0.49	.627
Age: phoneme /a/ vs. /ʃ/	0.02		0.03	0.59	.558
Age: phoneme /a/ vs. /s/	0.00		0.02	−0.06	.949
Age: phoneme /a/ vs. /t/	0.01		0.01	1.12	.263
**MCI TD vs. SSDs**					
**Predictor**	**Estimate**	***SE***	***df***	***t* value**	***p* value**
(Intercept)	3.55	0.08	30.21	44.69	< .001*
Age	−0.01	0.00	30.90	−2.83	.008*
Error (correct vs. error)	0.71	0.38	52.09	1.85	.069
Phoneme /a/ vs. /l/	−0.18	0.21	27.93	−0.86	.398
Phoneme /a/ vs. /ɹ/	1.02	0.27	44.05	3.84	< .001*
Phoneme /a/ vs. /ʃ/	0.26	0.25	34.44	1.02	.313
Phoneme /a/ vs. /s/	−0.65	0.21	42.25	−3.14	.003*
Phoneme /a/ vs. /t/	−0.55	0.19	24.61	−2.84	.009*
Age: error (correct vs. error)	0.00	0.00	43.04	−0.54	.592
Error (correct vs. error): phoneme /a/ vs. /l/	−1.66	0.85	73.01	−1.95	.055
Error (correct vs. error): phoneme /a/ vs. /ɹ/	−2.36	0.83	74.01	−2.83	.006*
Error (correct vs. error): phoneme /a/ vs. /ʃ/	−1.32	0.81	80.85	−1.63	.108
Error (correct vs. error): phoneme /a/ vs. /s/	−1.26	0.79	69.04	−1.60	.115
Error (correct vs. error): phoneme /a/ vs. /t/	0.01	0.01	25.82	0.60	.553
Age: phoneme /a/ vs. /l/	0.01	0.01	28.17	0.91	.369
Age: phoneme /a/ vs. /ɹ/	< 0.001	0.01	23.10	−0.40	.693
Age: phoneme /a/ vs. /ʃ/	< 0.001	0.01	25.39	0.34	.736
Age: phoneme /a/ vs. /s/	3.55	0.08	30.21	44.69	< .001*
Age: phoneme /a/ vs. /t/	−0.01	0.00	30.90	−2.83	.008*

*Note.* Results for predictors that are significant at the *p* < .05 level are bolded and marked with an asterisk. NINFL = Number of INFLections; MCI = modified curvature index; TD = typically developing; SSDs = speech sound disorders; PCC = percentage consonants correct; *SE* = standard error; *df* = degrees of freedom.

In addition, the model also suggests that there was a significant effect of age. Increased age was associated with lower tongue shape complexity for correct productions. In addition, similar to the findings for typically developing children, correct /ɹ/ productions had overall higher MCI and NINFL than /a/, while correct /t/ productions had lower NINFL and MCI than /a/. Correct /s/ productions had lower MCI than /a/. There was a significant interaction between age and phonemes /s/ and /t/ relative to /a/. The higher the age, the higher the tongue shape complexity for correct /s/ and the lower the tongue shape complexity for correct /t/ relative to /a/. The right panel in Figure 1 illustrates that this result needs to be interpreted with consideration of the decline in tongue shape complexity of /a/ as the children's ages increased. The positive interaction between age and phoneme /s/ relative to /a/ is actually a less steep decline in the tongue shape complexity of /s/ compared to /a/ and is not an overall increase.

## Discussion

This study set out to test the effects of a speech sound disorder diagnosis, age, consonant type, and perceptual accuracy on children's tongue shape complexity during speech, as measured by NINFL and MCI. Regarding the first research question, there was no evidence that children with speech sound disorders and typically developing children differ in NINFL, even when considering age and consonant. However, using MCI as a measure of tongue shape complexity revealed that children with speech sound disorders tended to have lower tongue shape complexity than typically developing children, as their age increased.

Regarding the second research question, there was mixed evidence for the relationship between age and tongue shape complexity. There was no significant relationship between age and NINFL, even considering diagnosis and consonant. However, when using MCI as a measure of tongue shape complexity, a higher age in both typically developing children and children with speech sound disorders was associated with lower tongue shape complexity.

Regarding the third research question, a significant relationship between the predictor and tongue shape complexity was found, again, in the MCI model but not in the NINFL model. When analyzing only the sample of children with speech sound disorders, /ɹ/ had significantly lower MCI when produced with a speech error than when it was correct, relative to /a/.

In addition to addressing the research questions, the results suggested that there were also significant differences in terms of tongue shape complexity between consonants. In both typically developing children and children with speech sound disorders, NINFL and MCI of /ɹ/ were higher than those of /a/, and NINFL and MCI of /t/ were lower than those of /a/. In typically developing children, both NINFL and MCI of /s/ were lower than those of /a/, keeping age constant. In the speech sound disorder group, MCI of /s/ was lower than that of /a/. Overall, there were more significant results that were consistent with theory for MCI than NINFL, similar to that in Kabakoff et al. (2022, 2023).

### Relationship Between Diagnosis and Tongue Shape Complexity

The first research question focused on the hypothesis that children with speech sound disorders might have more undifferentiated lingual gestures and difficulties with speech motor planning or control, compared to children without the diagnosis, resulting in lower NINFL and MCI across consonants. It was hypothesized that the difference between typically developing children and children with speech sound disorders might manifest more in older children and in consonants that require a higher level of tongue shape complexity. There was some support for this hypothesis using the MCI measure and when accounting for age. With an increase in age, children with speech sound disorders had lower tongue shape complexity compared to typically developing children across consonants. According to Figure 1, children with speech sound disorders showed a much more uniform pattern of decline of tongue shape complexity as age increased across all consonants under investigation, while in the typically developing children, there was a much more variable pattern.

The greater variability that can be observed in the plotted results for the typically developing group may be linked to the fact that the typically developing children were asked to produce only one token of the consonants, while the children with speech sound disorders were asked to produce multiple repetitions. According to the law of large numbers, a higher number of measurements would produce an average closer to the true mean and reduce the influence of random variation, which is disproportionately affecting smaller samples (Dekking et al., 2005). Hence, it is possible that the difference in tongue shape complexity observed here between typically developing children and children with speech sound disorders may change when more repetitions per child are collected. Future studies need to consider eliciting multiple repetitions per participant.

Another factor that may have influenced the results is that the older speakers in this sample of children with speech sound disorders may have had overall higher disorder severity than the younger children with speech sound disorders, leading to lower tongue shape complexity across consonants, explaining the lower MCI results. The diagnosis of persistent speech sound disorder is given to children who have not acquired the phonemes of their language by the age of 8 years (Wren et al., 2016), and research suggests that some speech sound disorders resolve by the age of 6.5 years (To et al., 2022). Hence, the children who remain in therapy would be likely to have more serious difficulties.

We ran a post hoc paired two-tailed *t* test between the children's age in months and their percentage consonants correct. The percentage consonants correct was calculated based on the perceptual accuracy results for the phonemes used in the other analyses. The test was significant, *t*(29) = 23.58, *p* < .001, but in the opposite direction to what was expected. Older children tended to have higher percentage consonants correct. Exploration of the data suggested that older children with speech sound disorder aged 9–12 years in this sample had errors of fewer different consonants than younger children aged 5–8 years. The older children only had errors of /s/ (*n* = 49 tokens), /ʃ/ (*n* = 41), and /ɹ/ (*n* = 32), while the younger children had errors of these consonants as well as /l/ and /t/. In Figure 3, /l/ and /t/ are associated with higher tongue shape complexity, while incorrect /ʃ/ and /ɹ/ are associated with lower tongue shape complexity, which could be contributing to the apparent decrease in tongue shape complexity with age. Despite the overall higher percentage consonants correct, the speech sound difficulties of the older children might still be considered severe due to their persistence.

According to Scobbie et al. (2023), the typical age of acquisition of Scottish English /ɹ/ in onset position is 6;0–6;5; that of /ʃ/ is 5;0–5;5; and that of /s/, /t/, and /l/ is 3;0–3;5. Three of the typically developing children in this study were younger than 7 years, and they could have still been refining the motor programs for /ɹ/ and /ʃ/, even if auditorily their productions were judged correct. It is possible that these typically developing participants contributed to the lack of significant MCI differences between typically developing children and children with speech sound disorders for any of the consonants.

The higher tongue shape complexity observed in the younger children with speech sound disorders (who also have lower percentage consonants correct) is consistent with the findings of Kabakoff et al. (2022). They reported that there was a negative relationship between perceptual accuracy and tongue shape complexity in the /ɹ/ of speakers with speech sound disorders before speech therapy intervention. They attributed this result to individual children having maladaptive response to unsuccessful previous speech treatment, leading to high complexity and low accuracy.

The results discussed in this section are in contrast to other studies, which report that typically developing children have higher tongue shape complexity, particularly for /ɹ/ (Kabakoff et al., 2021; Preston et al., 2019). In this study, there was no significant effect of a speech sound disorder diagnosis for the realization of /ɹ/, but a difference was observed across consonants, when age increased.

### Relationship Between Age and Tongue Shape Complexity

The second research question focused on the effect of age in typically developing children. It was predicted that as children grow, they increase their tongue shape complexity as their oral cavity increases in size relative to their tongue (Bosma, 1963), particularly in consonants that require more inflections.

However, the opposite relationship was observed. There was a significant effect of age in the typically developing and the speech sound disorder group for MCI. The pattern is similar to the findings of Kabakoff et al. (2021), who observed decreasing NINFL for /t/ with the increase in age in typically developing speakers. However, the results of the two studies are not directly comparable: Kabakoff et al. (2021) speakers' ages varied between 4;0 and 6;3, while this study focuses on the range of ages 5;0–12;11.

Despite the overall effect of age across consonants in the typically developing group, the raw data plot in Figure 1 reveals that the decrease in MCI is not universal for all consonants. While there were no significant interactions between age and individual phonemes, visual inspection of the plot suggests that the tongue shape complexity of /ɹ/, /s/, and /ʃ/ tended to decrease with the increase in age, while /a/ remained stable and /t/ and /l/ increased. The different patterns of development do not seem straightforwardly linked to the tongue shape complexity predicted by consonant hierarchies. For example, the increasing pattern is observed both for /t/, requiring a simpler gesture, and for /l/, one of the most differentiated consonants in English (Kabakoff et al., 2023). The decrease of /s/ and /ʃ/ is consistent with the qualitative differences between children and adults seen in the work of Zharkova (2013). In the context of the null interactions between age and phoneme, it is also possible that the observations made based on the left panel of Figure 1 are random variation.

This study failed to replicate the result of the work of Kabakoff et al. (2021), where /t/ in the typically developing children decreased in complexity with the increase in age. However, there was a significant interaction between age and correct realizations of phonemes /s/ and /t/ relative to /a/ in the speech sound disorder group. In the context of the right panel of Figure 1, these results were interpreted as a steeper decline in tongue shape complexity for /t/ relative to /a/ and for /a/ compared to /s/, as age increased. Hence, the result of the work of Kabakoff et al. (2021) was replicated in the correct productions of children with speech sound disorders. Notably, the group with speech sound disorders produced multiple repetitions of the consonants, unlike the typically developing children. The second research question predicted that increase in age will be associated with an increase in tongue shape complexity, particularly for the consonants requiring more complex gestures. The evidence against that comes from the negative effect of age on tongue shape complexity in the typically developing and the speech sound disorder group. Yet, the negative interactions between age and /t/ in the speech sound disorder group and in the typically developing children in the work of Kabakoff et al. (2021) involve a consonant expected to have simpler tongue shape complexity.

The only other study that has considered potential effects of maturation on tongue shape complexity is that of Kabakoff et al. (2023), who report data for NINFL and MCI for both adults and children. They were not able to compare their respective tongue shape complexities quantitatively because of the different elicitation tasks used for each group. However, they reported that the adults had overall higher NINFL than the children (up to 6 years), particularly for the liquids, consistent with the theoretical expectations. This observation receives some support in this study, as /l/ appears to increase in MCI with age, but /ɹ/ appears to slightly decrease, while still maintaining a relatively high level of complexity.

This lack of replication could, first, be the result of a nonlinear relationship between age and tongue shape complexity. It is possible that the anatomical changes in children, combined with the maturation of oral motor skills, may interact to lead to variable tongue shape complexity realization of each consonant at different time points. Second, the lack of replication can also be linked to the seemingly larger variability of consonant trajectories in the typically developing group, addressed in the previous section. This would suggest that a new study is required, which systematically investigates the typical development of tongue shape complexity across children of different ages, using multiple repetitions per token.

### Relationship Between Perceptual Accuracy and Tongue Shape Complexity

The effect of perceptual accuracy of each phoneme token was investigated within the speech sound disorder group. Initially, it was predicted that incorrect realizations of complex consonants would be linked to lower tongue shape complexity because of the higher likelihood of undifferentiated lingual gestures. However, that was not observed consistently in the present sample. Lower MCI was associated with incorrect compared to correct realizations of /ɹ/ relative to a reference /a/. Figure 3 illustrates this finding and also a tendency for incorrect /ʃ/ to have lower MCI than correct /ʃ/. This was not observed for any other consonants or with NINFL as a measure of tongue shape complexity.

This result is consistent with the findings of Preston et al. (2019) and of Kabakoff et al. (2021), who found an effect of accuracy (across typically developing speakers and speakers with speech sound disorders) on /ɹ/ for NINFL. They found no effect of accuracy on either /ɹ/ or /l/ for MCI. In addition, Kabakoff et al. (2022) report a positive relationship between perceptual accuracy and tongue shape complexity in /ɹ/ after children received speech therapy. This relationship was detected using MCI but not NINFL. This study adds to mounting evidence that incorrect realizations of /ɹ/ are likely to involve lower tongue shape complexity than accurate realizations.

There could be several explanations for the nonsignificant interactions between perceptual accuracy and the other consonants. First, it is possible that some consonants were produced incorrectly involving variable levels of complexity depending on the error type, meaning that a single sound could have been produced with an undifferentiated lingual gesture on one occasion and with inappropriately high number of inflections on another. Second, this potential variability, combined with the fact that different children did not always produce the same consonants incorrectly, would mean that there was too little power to detect a linear effect of inaccuracy in either direction. Future studies, involving larger samples of participants, could investigate the effect of error type on tongue shape complexity. It is also worth noting that common errors affecting the consonants under investigation, /l/, /s/, and /ʃ/, may involve the lateral parts of the tongue. Changes in lateral tongue movement would not be visible in a midsagittal ultrasound recording.

This study reports lower MCI in children with speech sound disorders compared to typically developing children, as well as lower MCI of incorrect compared to correct /ɹ/ productions. These results add to the evidence that supports speech motor control difficulties in children with idiopathic speech sound disorders (Cleland et al., 2017; Friel, 1998; Gibbon, 1999; Gibbon et al., 1993).

### Limitations and Future Directions

This study has several limitations. Firstly, the low sample size means that there are few individuals per age and variable representation of incorrect consonants across the speakers. The observed results might change if the same models are applied to a larger data set. Secondly, the use of highly controlled utterances (multiple repetitions of vowel–consonant–vowel syllables) was preferred considering the small sample size, as it controls for coarticulation effects and allows for a larger uniform speech sample. However, it is unclear whether the current results are generalizable to a more ecologically valid sample. For example, in the work of Kabakoff et al. (2021), children produced consonant–vowel–consonant real words, which somewhat limits comparisons between the two studies. Thirdly, the children with speech sound disorders in this sample have varying presentations and comorbidities. It is possible that different subgroups have different levels of difficulty with lingual differentiation. However, it was not feasible to investigate this with the existing data set, given the low numbers per subgroup. It is also possible that some children with speech sound disorders had differences in anatomy, dentition, and orofacial functioning from the typically developing group that have been undetected by ultrasound technique and not reported by their speech and language therapist. Future research on tongue shape complexity can also investigate the effect of speech sound disorder subtype and anatomical characteristics on tongue shape complexity. Third, it was observed that older children with speech sound disorders had systematically lower percentage consonants correct than younger children, which is a limitation of the cross-sectional design. Longitudinal research is needed for a more informative investigation into the relationship between age, percentage consonants correct, and tongue shape complexity.

The results reported here add to the series of recent reports about the use of NINFL and MCI in studying tongue shape complexity in children with speech sound disorders. In the work of Kabakoff et al. (2022), NINFL performs better qualitatively but MCI is the more sensitive measure quantitatively. In addition, in the work of Kabakoff et al. (2022), MCI detects the theoretically predicted relationship between /ɹ/'s tongue shape complexity and accuracy, as well as the relationship between tongue shape complexity and somatosensory acuity, while NINFL did not detect any of these relationships. This is contrary to earlier findings in the works of Kabakoff et al. (2021) and Preston et al. (2019). Kabakoff et al. (2022) argue that might be the result of MCI's sensitivity to the size of the local curves. Hence, retroflex tokens might have high MCI because of one large local curve but relatively lower NINFL because of a smaller total number of curves. Thus, if more retroflex shapes are present in a sample, MCI would be the more sensitive measure (Kabakoff et al., 2022). By the same logic, MCI would be more sensitive to complexity differences in other consonants that require lower number of inflections than a bunched /ɹ/.

This was borne out in this study, as MCI was more sensitive than NINFL. MCI detected more differences between lingual consonants and the reference in the typically developing group and speech sound disorder group compared to NINFL. In addition, MCI detected an effect of age on tongue shape complexity, an interaction between age and diagnosis, and an interaction between accuracy and phoneme. More work is required to replicate and expand on these results, but one potential future outcome might be that MCI and NINFL are applied to study different consonants.

## Conclusions

This study provides an in-depth investigation of tongue shape complexity, using the NINFL and MCI measures. These measures were used to investigate the effects of speech sound disorder diagnosis, age, consonants, and perceptual accuracy of consonants on tongue shape complexity across children. The results indicated that as children's age increased, tongue shape complexity decreased more for children with speech sound disorders than for typically developing children. The initial prediction that typically developing children would have higher tongue shape complexity than children with speech sound disorders was supported, but only when accounting for age. The prediction that an increase in age would lead to an increase in tongue shape complexity was not supported: Unexpectedly, increase in age was associated with a decrease of MCI across consonants for both typically developing children and children with speech sound disorders. Lastly, /ɹ/ tokens categorized as incorrect had significantly lower tongue shape complexity than correct ones, relative to /a/ for children with speech sound disorders, which is consistent with findings from other studies.

The results corroborate previous findings that children with speech sound disorders have subtle motor difficulties. The results also suggest that speech motor difficulties may manifest as low tongue shape complexity, depending on the consonant, the speaker's age, and perceptual accuracy.

## Data Availability Statement

The numerical data set and the statistical code are available at https://osf.io/9cwfg/?view_only=d2856a03a95d44e192cdad1cf56bb4e6. The ultrasound recordings are available at https://ultrasuite.github.io/.

## Footnotes

1
In ultrasound imaging, the point of maximum contact is determined by annotating the position of the tongue that is highest relative to its preceding and following positions and relative to the position of the palate, which can be inferred from a recording of a swallow (Kochetov, 2020).

2
Note that the type of ultrasound used for recordings should not affect the NINFL measure, as long as the tongue curve is visible. Kabakoff et al. (2021) argue that NINFL and MCI can be measured independently of spatial orientation and size, which may vary with different depth settings, as they are based on curvature measures (Ménard et al., 2012; Stone, 2005), and within these static measures, even a difference of up to 48 ms between the ultrasound image and auditory signal would still result in a sufficiently accurate frame identification. In the present sample, this difference would be about 10 ms.

3
It should be noted that the NINFL formula in Articulate Assistant Advanced is based on the work of Preston et al. (2019), whereas Kabakoff et al. (2021) used an updated version. They applied a low-pass filter on the signed curvature (0.075) and changed the trim threshold to 0.002 based on a heuristic procedure to match the NINFL output to results from visual inspection of selected representative curves.

## Appendix A

### Demographic Information for TD Participants

**Table:**

Participant	Sex	Age	BPVS SS
02TD	M	11;10	94
03TD	M	11;9	115
04TD	F	10;5	94
06TD	M	9;11	113
07TD	F	9;10	93
08TD	F	9;4	86
09TD	M	8;8	129
10TD	F	6;9	115
11TD	F	11;3	102
12TD	M	8;1	119
13TD	M	6;8	120
14TD	F	11;7	94
15TD	M	12;4	104
16TD	M	7;11	117
17TD	F	12;10	121
18TD	M	10;8	116
19TD	F	7;2	98
20TD	M	12;6	105
21TD	M	11;1	108
22TD	F	5;8	92
23TD	M	7;2	99
24TD	F	12;2	85
25TD	F	8;7	85
26TD	M	10;0	104
27TD	F	7;11	117
28TD	M	11;5	76
29TD	F	7;3	107
30TD	F	7;11	84
31TD	F	10;5	72

*Note.* Age reported in years;months. TD = typically developing; BPVS SS = British Picture Vocabulary Scale Standard Score; M = male; F = female.

## Appendix B

### Demographic Information for Participants With SSDs

**Table:**

Speaker	Sex	Age	Speech disorder reported by SLT	Additional information reported by SLT
LN01	M	8;2	Phonological disorder	–
LN02	F	6;3	Articulation disorder	–
LN03	M	9;2	Articulation disorder	–
LN04	M	10;9	Articulation disorder	–
LN05	M	5;9	Articulation disorder	Low muscle tone
LN06	F	9;0	–	
LN07	M	6;3	Motor speech disorder	Poor coordination
LN08	F	6;3	Phonological disorder	–
LN09	M	5;11	Inconsistent phonological disorder or CAS	–
LN10	M	6;4	Inconsistent phonological disorder	–
LN11	F	5;2	Diagnostic process ongoing	–
LN12	M	8;2	Motor speech disorder	Born at 31 weeks. Fine motor difficulties
GR01	M	9;1	Articulation disorder	
GR02	M	9;2	Inconsistent speech sound disorder, query CAS	DLD, swallowing difficulties
GR03	M	8;11	Residual speech sound errors	Previous surgery for anterior tongue tie
GR04	M	6;2	Articulation disorder	–
GR05	M	7;5	Articulation disorder	–
GR06	M	7;5	Articulation and phonological disorder with possible coordination difficulties	Difficulties with grammar
GR07	M	7;11	Articulation disorder	–
GR08	M	6;0	Possible CAS	–
GR09	M	12;11	Phonological disorder	Global developmental delay
GR11	M	8;4	Articulation disorder	Bilateral conductive hearing loss, wearing hearing aids
GR12	F	11;6	Motor speech disorder	ADHD and learning difficulties
GR13	F	11;2	Articulation disorder	–
GR14	M	10;10	Articulation disorder	–
GR15	F	7;4	Articulation disorder	–
GR17	M	8;5	Articulation disorder	–
GR18	M	7;10	Articulation disorder	Asthma
GR19	F	7;8	Articulation disorder	–
GR20	M	7;8	Articulation disorder	–

*Note.* Age reported in years;months (Sugden & Cleland, 2022, p. 7). LN and GR are confidential data collection codes. The dashes mean no additional information reported. SSDs = speech sound disorders; SLT = speech and language therapist; M = male; F = female; CAS = childhood apraxia of speech; DLD = developmental language disorder; ADHD = attention-deficit hyperactivity disorder.
